# Transcriptional and Translational Regulatory Responses to Iron Limitation in the Globally Distributed Marine Bacterium *Candidatus* Pelagibacter ubique

**DOI:** 10.1371/journal.pone.0010487

**Published:** 2010-05-05

**Authors:** Daniel P. Smith, Joshua B. Kitner, Angela D. Norbeck, Therese R. Clauss, Mary S. Lipton, Michael S. Schwalbach, Laura Steindler, Carrie D. Nicora, Richard D. Smith, Stephen J. Giovannoni

**Affiliations:** 1 Molecular and Cellular Biology Program, Oregon State University, Corvallis, Oregon, United States of America; 2 Department of Microbiology, Oregon State University, Corvallis, Oregon, United States of America; 3 Biological and Computational Sciences Division, Pacific Northwest National Laboratory, Richland, Washington, United States of America; Universidad Miguel Hernandez, Spain

## Abstract

Iron is recognized as an important micronutrient that limits microbial plankton productivity over vast regions of the oceans. We investigated the gene expression responses of *Candidatus* Pelagibacter ubique cultures to iron limitation in natural seawater media supplemented with a siderophore to chelate iron. Microarray data indicated transcription of the periplasmic iron binding protein *sfuC* increased by 16-fold, and iron transporter subunits, iron-sulfur center assembly genes, and the putative ferroxidase rubrerythrin transcripts increased to a lesser extent. Quantitative peptide mass spectrometry revealed that *sfuC* protein abundance increased 27-fold, despite an average decrease of 59% across the global proteome. Thus, we propose *sfuC* as a marker gene for indicating iron limitation in marine metatranscriptomic and metaproteomic ecological surveys. The marked proteome reduction was not directly correlated to changes in the transcriptome, implicating post-transcriptional regulatory mechanisms as modulators of protein expression. Two RNA-binding proteins, CspE and CspL, correlated well with iron availability, suggesting that they may contribute to the observed differences between the transcriptome and proteome. We propose a model in which the RNA-binding activity of CspE and CspL selectively enables protein synthesis of the iron acquisition protein SfuC during transient growth-limiting episodes of iron scarcity.

## Introduction

The importance of iron as a nutrient in the oceans was first recognized by Martin [1] and later experiments verified that iron limits primary production over broad regions of the marine environment [2]–[4]. A variety of biological processes such as photosynthesis, N_2_ fixation, methanogenesis, respiration, oxygen transport, gene regulation, and DNA synthesis all depend on iron-containing proteins [5]. In pelagic surface waters, planktonic communities must cope with iron concentrations that average just 70 picomolar [6]. The inhibitory effect that this has on growth was most clearly illustrated by a series of iron fertilization experiments in which iron was added to large swaths of the ocean, resulting in a marked increase in nutrient utilization [2], [3], [7].

Bacteria commonly have specialized systems for responding to iron limitation. Genes for iron uptake and utilization are primarily regulated by the Fur protein [8], [9]. When complexed with Fe(II) cations, Fur binds the “Fur box” recognition sequence, which is made of several GATAAT hexamers [10]–[14]. In some bacteria, this single transcription factor can directly repress or activate more than 100 genes in response to iron scarcity [9]. Irr is a similar transcription factor that couples intracellular heme levels to expression of many different iron-related pathways [15]–[21]. Small RNAs [22]–[25] and mRNA-binding proteins [26], [27] can also regulate nonessential iron-utilizing proteins at the post-transcriptional level by selectively targeting their transcripts for degradation. To improve their chances of encountering Fe(III), many bacteria secrete siderophores [28]–[30]. These chelating agents help dissolve the poorly soluble particles and sequester them in a form that is unusable by competing microorganisms. Due to the spontaneous reactivity of iron ions, cells often encapsulate these atoms inside containers made of ferritin proteins to better modulate redox reactions [31], [32].

*Candidatus* Pelagibacter ubique was selected as an iron limitation model for two reasons. First, this alphaproteobacterium is regularly the most numerically abundant microorganism in surveys of marine microbial diversity. Second, its proteome of just 1,354 genes is possibly the simplest of any free-living heterotrophic organism [33]. *Ca.* Pelagibacter ubique's genome encodes Fur and Irr, but not ferritin or siderophore-related proteins, raising questions about how or if this bacterium can cope with iron stress. Investigating how this organism's relatively small genetic repertoire produces thriving populations in the variable ocean environment has been impeded by the lack of a genetic system able to create knockouts or other genetic modifications. Thus, observing how the entire transcriptome or proteome changes in response to growth conditions has become a primary approach for elucidating metabolic and regulatory schemes [34]–[36]. A comparison of cultures in exponential and stationary phase did not reveal a major remodeling of the proteome nor evidence of a global regulatory mechanism [34], suggesting that this organism may continue to benefit from temporary nutrient availability regardless of overall cellular activity. That study, along with a follow-up using a metaproteomics approach on environmental samples [35], found *Ca.* Pelagibacter ubique's proteome to be consistently composed of an unusually high proportion of transport-related proteins.

Arguably the most important characteristic of organisms is their ability to express the right proteins in the right amounts at the right times. The interplay between stimuli, sensors, and regulators precisely optimizes the combination of mRNA transcripts and protein products present in the cell. Several known and putative transcriptional regulators have been identified in the *Ca.* Pelagibacter ubique genome, as well as cis-acting riboswitches capable of modulating mRNA translation based on the concentration of particular metabolites. This method of decoupling production of mRNA from protein synthesis has been found on glycine metabolism genes in *Ca.* Pelagibacter ubique [37] and sequence motif searches [38] found additional candidate riboswitches for s-adenylmethionine [39], [40] and thiamine pyrophosphate [40], [41]. Meyer and colleagues also identified homologs to ribosomal proteins capable of regulating their own translation, as well as regions in the genome with riboswitch-like characteristics but lacking homologous annotated motifs. One of these putative structural RNA regions is located immediately upstream of the *sfuA–C* operon, which encodes an iron-acquisition system. Post-transcriptional regulation schemes allow the cell to conserve amino acids while still rapidly providing ephemeral enzymes. The success of these characteristics are evidenced by direct observation; *Ca.* Pelagibacter ubique is the most abundant heterotroph in the oceans, accounting for one-third of surface water bacteria [42], [43] and consuming up to half of some dissolved organic matter compounds [44].

Combining transcriptomic and proteomic data offers a perspective on cellular activity that cannot be obtained from either method individually. Numerous studies have shown that changes in the transcriptome poorly correlate with changes to the proteome, except for very highly expressed genes [45]–[48]. Although much of the disparity between these two types of datasets has been attributed to measurement inaccuracy [49] and differences in protein degradation rates [50], some studies have revealed systematic post-transcriptional regulatory schemes. For instance, in the eukaryotic protozoan *Plasmodium falciparum*, mRNAs were often upregulated an entire life phase before the one in which the encoded protein was needed [51]. Additionally, translation in *Escherichia coli* was found to be partially regulated by mRNA secondary structure [52]. Therefore, it is evident that the transcriptome does not necessarily represent the current state of the proteome, but is rather a mixture of transcripts being actively translated and others that are standing by, awaiting activation by post-transcriptional regulatory mechanisms. This study integrates both transcriptomic and proteomic analyses in order to attain a more complete understanding of the cellular response to iron limitation in *Ca.* Pelagibacter ubique. The results strongly suggest that transcription and translation are not always tightly coupled in this bacterium.

## Results

### Reaction to the Siderophore

**Figure 1:**
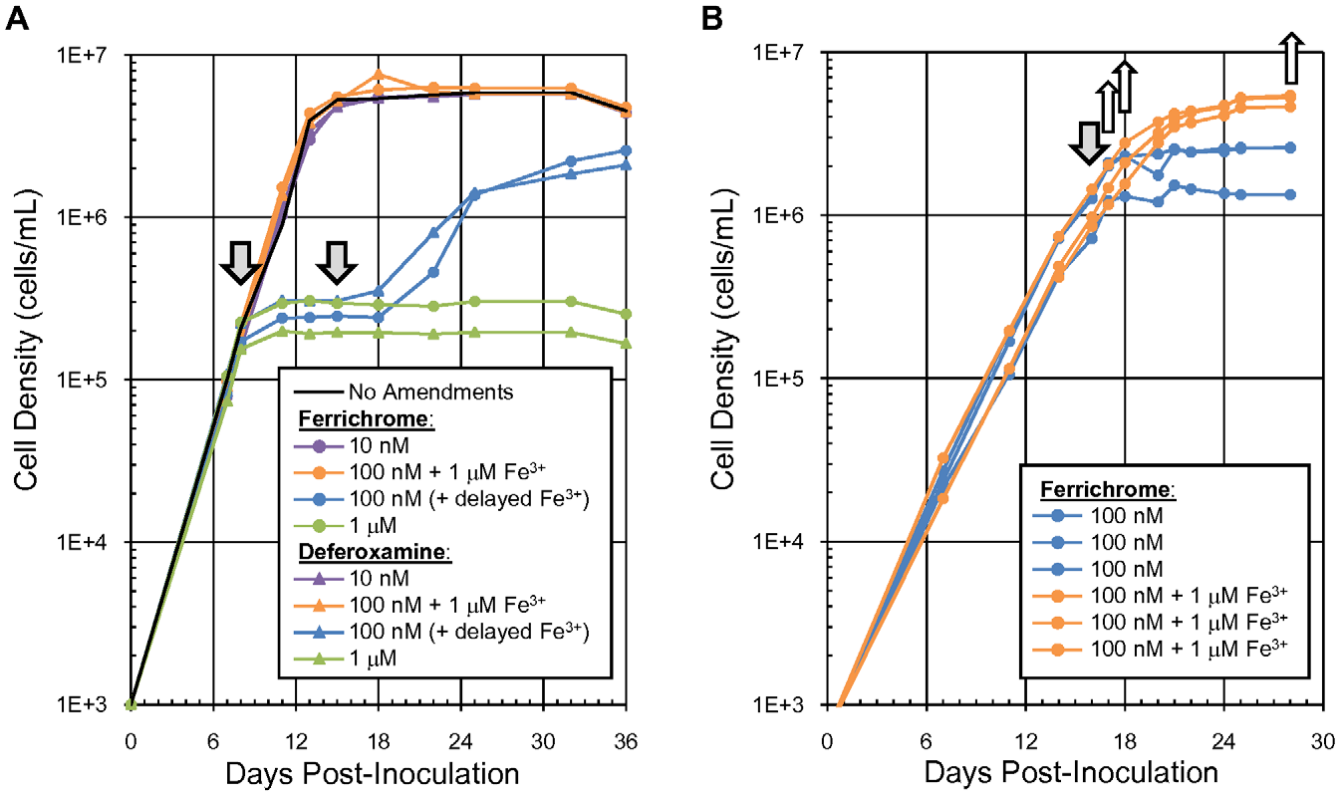
Growth of *Candidatus* Pelagibacter ubique cells was arrested by iron-sequestering siderophores. (A) Cell densities observed during a pilot experiment to test the effect of the two siderophores ferrichrome and deferoxamine mesylate salt at varying concentrations on the growth of *Candidatus* Pelagibacter ubique HTCC1062. The first arrow indicates the introduction of siderophore/iron as described by the legend. The second arrow indicates the delayed 1 µM iron additions parenthetically noted in the legend. (B) Cultures for harvesting were grown in six 20 L carboys. The first arrow indicates the introduction of siderophore/iron as described by the legend. Proteins and mRNA were analyzed on the dates indicated by the unfilled arrows: microarray samples were taken from cultures on days 17, 18, and 28; proteomic samples were taken on days 18 and 28.

Two iron-sequestering siderophores were tested on cultures of *Ca.* Pelagibacter ubique to determine their feasibility for creating iron-limiting conditions. Ferrichrome (Sigma #F8014) and deferoxamine mesylate salt (Sigma #D9533) were both found to arrest batch culture growth within 1/3 of a doubling – an inhibition which could be reversed by addition of iron (Figure 1A). Sufficient bioavailable iron was present in the natural seawater media collected from the Oregon coast to enable cultures to grow when supplemented with 10 nM siderophore, but not when supplemented with 100 nM siderophore.

### Microarrays

Six 20 L carboys inoculated with *Ca.* Pelagibacter ubique were grown to near-maximum density, then randomly selected for treatment with either ferrichrome or ferrichrome plus excess iron (Figure 1B). To measure the amount of messenger RNA transcripts present in cells, mRNA from each carboy was hybridized to separate microarray chips containing probes for all *Ca.* Pelagibacter ubique genes. Microarray data was deposited in the NCBI GEO database under accession number GSE20962. Of the three time points where mRNA abundance was measured, the greatest difference in expression of known iron-related genes was observed 24 hours after the siderophore amendment. Table 1 lists the 23 transcripts that were expressed at least 50 percent higher in the iron-limited culture relative to the control. Two-thirds of the genes in this list come from two operons: the first containing iron-sulfur center assembly proteins including *sufA–E* and the second made up of iron uptake proteins such as *sfuA–C*. The four other genes with a known function are: rubrerythrin, an iron-binding protein that is postulated to act as a ferroxidase for converting Fe(II) to Fe(III), *hslU* and *hslV* which together form a protease complex, and dimethylglycine dehydrogenase – an enzyme that is necessary for converting betaine to glycine.

**Table 1:** All 23 *Ca.* Pelagibacter ubique mRNA transcripts that were at least 50 percent more abundant in the iron-limited cultures compared to the control cultures, 24 hours after addition of an iron-chelator.

Locus ID	Gene	Description	Ratio^a^	P Value^b^	Cluster
SAR11_0144		Conserved hypothetical protein	1.54	0.001	Early
SAR11_0333 •	*hslV*	ATP-dependent protease: peptidase	1.63	0.000	Stat.
SAR11_0334 •	*hslU*	ATP-dependent protease: ATP-binding	1.59	0.035	
SAR11_0399	*rbr*	Rubrerythrin, hyp. ferroxidase (Fe^2+^→Fe^3+^)	1.57	0.025	
SAR11_0738 •	*sufA*	Transcriptional regulator	1.76	0.062	Stat.
SAR11_0739 •	*sufB*	Cysteine desulfurase activator complex	2.00	0.009	Early
SAR11_0740 •	*sufC*	FeS assembly ATPase	1.80	0.017	Early
SAR11_0741 •	*sufD*	FeS assembly protein	2.29	0.019	Early
SAR11_0742 •	*csdB*	Selenocysteine lyase chain A	2.44	0.046	Early
SAR11_0743 •	*sufE*	Putative NifU-like protein	2.24	0.004	Late
SAR11_0744 •	*paaD*	Phenylacetic acid degradation protein	2.00	0.001	Early
SAR11_0745 •	*hesB*	HesB protein	1.95	0.000	Early
SAR11_0785		Conserved hypothetical protein (DUF952)	1.52	0.051	Late
SAR11_1233 •		Domain of unknown function (DUF931)	3.36	0.001	Early
SAR11_1235 •	*azlC*	AzlC protein	2.14	0.016	Early
SAR11_1236 •	*sfuA*	Iron(III) ABC transporter: ATP-binding	4.99	0.000	Early
SAR11_1237 •	*sfuB*	Iron(III) ABC transporter: permease	10.36	0.000	Early
SAR11_1238 •	*sfuC*	Iron(III) ABC transporter: periplasmic	16.00	0.003	Early
SAR11_1239 •		Unknown protein	2.47	0.006	Late
SAR11_1240 •	*aceA*	Isocitrate lyase	1.58	0.091	Late
SAR11_1242 •		Transcription regulator	1.60	0.054	Late
SAR11_1253	*dmgdh*	Dimethylglycine dehydrogenase	1.54	0.054	Late
SAR11_1279		Unknown membrane protein	1.56	0.056	

**Figure 2:**
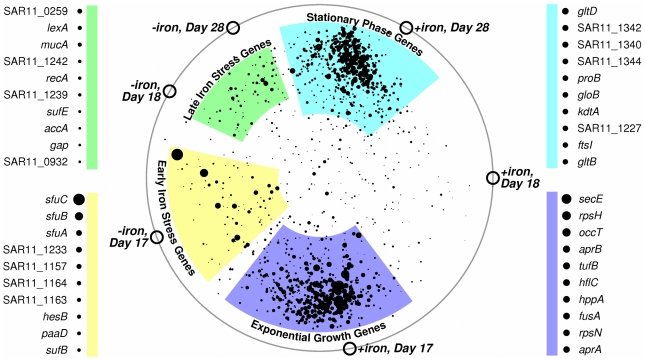
Genes transcribed during iron limitation were different from stationary phase genes. The four clusters indicate up-regulation of similar condition-specific mRNA. Symbols for each microarray sample (open circles) were manually positioned on a circle according to each sample's iron availability and growth rate. Genes were “attracted” to the samples in which they were most abundant. Larger points indicate genes with larger condition-to-condition variation; a key for the 10 largest points in each cluster is provided. The complete list of gene locations for this graph can be found in Supplementary Table S1.

**Seventy-eight percent of these genes are found in Figure 2's early and late iron stress clusters. Bullet points in the first column indicate contiguous loci.**

a Average fluorescence of three replicates, (iron limited culture/iron replete culture).

b Result of a two-tailed Student's t-test comparing the three biological replicates for each treatment.

A modified radial coordinate visualization plot (Figure 2) of the microarray data shows four distinct clusters of genes: exponential growth, stationary phase, early iron stress, and late iron stress. As detailed in Supplementary Table S1, the early iron stress cluster is dominated by genes from three genomic loci: the *sfu* iron uptake operon, the *suf* iron-sulfur center assembly operon, and the functionally unclear loci SAR11_1157, SAR11_1158, SAR11_1163, and SAR11_1164. The late iron stress cluster also contains different genes that are located in or adjacent to the *sfu* and *suf* operons, but is better characterized by *lexA*, *recA*, and *mucA* – three genes involved in the SOS response. The iron response regulators *fur* and *irr* cluster with stationary phase genes, indicating that the abundances of these two transcripts are more affected by stationary phase than by iron limitation.

### Proteomics

Cellular protein fractions from each treatment were isolated and digested before being separated with liquid chromatography and injected into a tandem mass spectrometer. An Accurate Mass and Time Tag library, developed previously [34], was used to make quantitative comparisons of the abundance of individual peptides between samples. This dataset is available at http://omics.pnl.gov/. Of the 216 proteins detected with high certainty in this study, 18 were observed to be at least 50% more abundant in the iron-limited cultures: four on day 18, and 17 on day 28 (Table 2). The proteins SfuC, CspL, and GroES were higher in the iron-limited cultures at both timepoints. The iron-binding SfuC is unique in that it was the only one of these 18 proteins to increase in both protein and mRNA abundance by at least 50%. CspL was originally annotated as a DNA-binding protein, however, similar proteins have been found to modulate the accessibility of mRNA binding sites by selectively melting secondary RNA structures [53]. The third protein, GroES, forms a complex with GroEL to mediate protein folding. Because the required GroEL subunit was much less abundant in iron-limited cultures, and since the three largest GroES peptide spectra (out of 9) were less pronounced in the iron-limited cultures, GroES may be a false positive. Mass spectrometry measurements did not reveal a signficant change in Fur or Irr abundance between treatments or timepoints.

**Table 2:** All 17 *Ca.* Pelagibacter ubique proteins that were at least 50 percent more abundant in the iron-limited cultures compared to the iron replete cultures, two and 12 days after addition of an iron chelator.

Day	Locus ID	Gene	Description	Ratio^a^	P Value^b^
18	SAR11_1238	***sfuC***	Iron(III) ABC transporter: periplasmic	11.41	0.000
18	SAR11_1274	***cspL***	DNA-binding cold shock protein	6.20	0.000
18	SAR11_0161	***groES***	Protein-folding chaperonin	2.01	0.028
18	SAR11_1062	*dapA*	Dihydrodipicolinate synthase	1.53	0.849
28	SAR11_1238	***sfuC***	Iron(III) ABC transporter: periplasmic	26.96	0.000
28	SAR11_1161	*sbcC*	ATPase involved in DNA repair	4.59	0.011
28	SAR11_0161	***groES***	Protein-folding chaperonin	3.59	0.008
28	SAR11_0601	*ftsH*	Metalloprotease	3.28	0.001
28	SAR11_1124	*rplL*	50S ribosomal protein L31	3.16	0.006
28	SAR11_0430	*aceF*	Dihydrolipoamide S-acetyltransferase	3.02	0.094
28	SAR11_0171		Rhodanese-related sulfurtransferase	2.74	0.002
28	SAR11_0791		Ring-cleaving dioxygenase	2.42	0.336
28	SAR11_1274	***cspL***	DNA-binding cold shock protein	2.27	0.437
28	SAR11_0235	*pdhD*	Dihydrolipoyl dehydrogenase	2.26	0.035
28	SAR11_0401		Conserved hypothetical protein	2.21	0.003
28	SAR11_0054	*pilA*	Pilin protein	2.16	0.020
28	SAR11_0727	*accB*	Acetyl-CoA carboxylase	2.03	0.301
28	SAR11_0987	*ppiB*	Peptidylprolyl isomerase	1.99	0.291
28	SAR11_0793		Unknown protein	1.74	0.128
28	SAR11_0599		Hypothetical protein	1.70	0.623
28	SAR11_0708	*acpP*	Acyl carrier protein	1.55	0.198

**Genes in bold were more abundant in the iron-limited cultures at both timepoints.**

a Average spectra height of at least three peptides, (iron limited culture/iron replete culture).

b Combined one-tailed Student's t-test comparing the three technical replicates for each treatment.

Iron-limitation had a marked impact on the overall proteome. Two days after addition of an iron-chelator, 181 of the 216 proteins were significantly (P≤0.05) less abundant in the iron-limited cultures relative to the control cultures. Using the same criteria, only 32 of the 216 proteins were found to significantly decrease in the control cultures between days 18 and 28 as *Ca.* Pelagibacter ubique cells entered stationary phase due to an unknown, non-iron, limitation.

### Comparing Changes in mRNA and Protein Abundances

**Figure 3:**
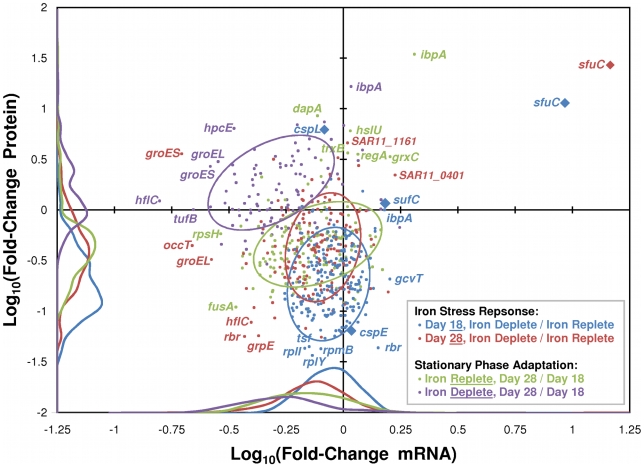
Protein abundances were largely decoupled from transcript abundances. The change in protein abundance versus the change in mRNA abundance was plotted for all *Ca.* Pelagibacter ubique genes that showed a significant (P< = 0.05) change in either measurement. Each color represents a different comparison between treatments or timepoints, with *R^2^* values of 0.11, 0.08, 0.09, and 0.02 respective to the legend's ordering. Large ellipses indicate clusters of the same colored points. Histograms on the low end of each axis further define the distribution of points. Points represented by a diamond are discussed at length in the text.

Aside from the highly expressed iron-binding protein SfuC, the abundances of individual proteins appeared to be independent of the amount of mRNA encoding them (Figure 3).

## Discussion

We are studying keystone microbial plankton species such as *Ca.* Pelagibacter ubique in culture to provide a basis for interpreting data emerging from molecular ecology studies. In an era of rapid environmental change, metagenomics, and allied technologies such as metaproteomics and metatranscriptomics, are being used to monitor the structure and health of natural ecosystems and to identify ecological processes that impact biogeochemistry. Interpretations of these data depend on understanding how complex cellular systems respond to environmental factors. We focused on a microorganism, Ca. Pelagibacter ubique, that produces the largest signal in most environmental studies of marine macromolecules, and a process, iron limitation, that impacts marine ecology on very large geographical scales.

### Upregulation of *sfuC* during iron limitation

The only gene to clearly increase in both mRNA and protein abundance during iron limitation was *sfuC*. This protein localizes to the periplasmic space and binds dissolved Fe(III) with high affinity. The SfuC-Fe complex associates with the ATPase (SfuA) and permease (SfuB) components of the tripartite ABC transporter complex to actively transport iron into the cell. The fact that *sfuA* and *sfuB* were not observed to increase in protein abundance is not wholly unexpected – SfuA–SfuB complexes only interact with iron-bound SfuC proteins, which are a very small fraction of the total SfuC pool in an iron-limited environment. Additionally, integral membrane proteins such as SfuB are particularly challenging to recover in proteomic studies because they are not readily soluble. This likely contributed to the complete absence of SfuB peptides in all mass spectrometry studies of *Ca.* Pelagibacter ubique to date.

The identification of *sfuC* expression as a readily quantifiable iron limitation marker is particularly useful for ecological surveys. As its name suggests, *Ca.* Pelagibacter ubique's genome, transcriptome, and proteome regularly dominate bacterial surveys throughout the pelagic environment. Future oceanographic studies seeking evidence of iron availability limiting bacterioplankton growth may use metatranscriptomic or metaproteomic analyses to assess the expression of *sfuC* in the local *Ca.* Pelagibacter ubique population.

### Transcriptome distinct from proteome

Protein abundance was generally uncorrelated with changes in mRNA abundance, suggesting that post-transcriptional mechanisms might be acting at the RNA level to suppress translation. As reviewed in the introduction, previous studies have shown that disparities between a cell's transcriptome and proteome are the norm rather than the exception. However, the observation that iron-related genes such as *sufA–E* increased in mRNA but not protein indicates that expression of these proteins are controlled at both the level of transcription and at the level of translation.

### Cold-shock proteins correlated with iron stress

**Figure 4:**
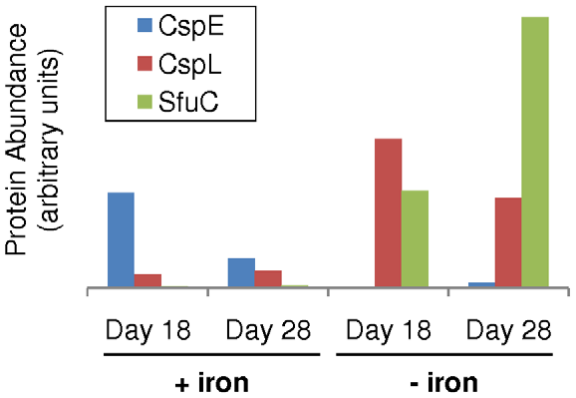
Translation of *Ca.* Pelagibacter ubique's cold shock and iron-binding genes are influenced by iron availability. The abundance of two *Ca.* Pelagibacter ubique cold shock proteins, CspE and CspL, and the iron-binding protein SfuC, appear to be correlated with iron availability (p-value of .02, .08, and 3e-79, respectively).

CspL was significantly more abundant in iron-limited cultures (Figure 4), leading us to closely examine the biological activity of this protein as well as the inversely expressed homolog CspE. The first discovered member of the cold-shock protein (CSP) family, *E. coli*'s CspA, is highly upregulated under cold stress; it is believed to associate with and melt double-stranded RNA complexes as a mechanism to prevent spurious stem loop structures from interfering with transcription and translation [54]–[59]. Despite their homology to CspA, many CSP variants are not cold-inducible, but rather are involved in regulating cellular processes [54], [60]–[62] and can even target their activity to specific RNA sequences [63]. A growing body of literature has described mRNAs which modulate their own expression via temperature- (RNA thermometer) or ligand-sensitive (riboswitch) secondary structures [64], [65]. Due to the episodic nature of iron deposition into ocean surface waters [66] and the resulting selective pressure favoring rapid response systems for this limiting nutrient [67], we speculate that *Ca.* Pelagibacter ubique CspE and/or CspL affects a reversible inhibition of translation by facilitating an mRNA secondary structure unfavorable for ribosome processing, thereby maintaining the transcriptome in a state of cell growth readiness during times of stress such as iron limitation.

This is the first report describing the general suppression of translation across the entire transcriptome of the cell. In this case, the apparent adaptive significance of protein synthesis suppression is related to urgent cellular requirements to acquire an essential nutrient. The model we propose to explain this phenomenon incorporates activity previously observed in cold-shock proteins, however, the essence of our model assigns cold-shock proteins a new systemic role in *Ca.* Pelagibacter ubique cells with the apparent result of focusing protein synthesis on transporters that target a missing essential nutrient. The validation of this model is beyond the scope of this study. Future work may more precisely identify interactions between cold-shock proteins and specific RNA motifs.

### Summary

Census information has left little doubt that *Ca.* Pelagibacter ubique plays its role in biological oceanography on a vast scale. To understand this role, we turned inward, investigating the mechanisms used by these cells to respond to a common form of nutritional stress. One motivation for this study can be described with a term borrowed from satellite remote sensing: the term “ground truth” was coined to describe the validation, by direct measurements, of remotely sensed observations. Metatranscriptomic and metaproteomic measurements are being widely adopted by microbial ecologists anticipating that these approaches will reveal the metabolic status of cells in microbial communities, providing information that can be extrapolated to interpret broader levels of ecosystem function. Essential to this vision is an understanding of how cells respond to environmental variables. Our findings indicate that the periplasmic iron binding protein *sfuC* is uniquely suitable for assessing the iron limitation status of *Ca.* Pelagibacter ubique cells. We anticipate that ecologists will use this data for interpreting the nutritional status of *Ca.* Pelagibacter ubique cells in nature.

This study, one of the few to simultaneously examine both transcriptional and translational responses in a bacteria cell, uncovered evidence suggesting that *cspL* might play a role in the cellular response to iron limitation. We offer the model that this protein controls translation in response to environmental conditions for a specific subset of genes present in the transcriptome. We hypothesize that this activity might serve an emergency function, limiting the synthesis of proteins to those that are critical for survival. This finding is consistent with previous reports of post-transcriptional regulation of the iron stress response in which a protein was found to facilitate the degradation of specific mRNAs which encoded nonessential iron-consuming pathways [26], [27].

Not only is *Ca.* Pelagibacter ubique one of the most successful cells known, it is also one of the simplest, giving it value as a model for understanding bacterial cell responses. Indeed, numerous new structural RNAs, some widely distributed among bacteria, have been discovered and described in *Ca.* Pelagibacter ubique [37], [38]. It is perhaps hubris to imagine that the concept of systems biology might one day be extended from the machinery of cells to the machinery of microbial ecosystems at work on the scale of oceans. But, if that vision has a chance, it will be by combining studies that cross scales and disciplines to understand the keystone species of the oceans.

## Materials and Methods

### Growth Media and Harvesting

Seawater was collected on 6/14/08 at the Newport Hydroline station NH5 (44°39.1′N, 124°10.6′W) from a depth of 10 m. The water was then filtered through a 0.2 µM filter, autoclaved, and sparged with CO_2_ for 24 hours followed by air for 24–48 hours as previously described [68], [69]. Immediately prior to inoculation with *Ca.* Pelagibacter ubique HTCC1062, the media was amended with 50 µM pyruvate, 50 µM glucose, 10 µM nitrogen, 1 µM methionine, 1 µM glycine, 1 µM phosphate, and vitamins. Cells were grown at 20°C (flasks) or 16°C (carboys) with intermittent light and sparging with air. On day 16, three 20 L control cultures were amended with 100 nM ferrichrome and 1 µM FeCl_3_, and three 20 L treatment cultures were amended with 100 nM ferrichrome only. On day 18, 8 L from each carboy was harvested. On day 28, the remaining ∼10L from each was harvested. Prior to each harvest, and on day 17, three 40 mL samples of culture were removed from each culture for microarrays. Water from the three replicate cultures were then combined and growth was arrested using 0.01g chloramphenical and 0.1 mL protease inhibitor cocktail II (CalBiochem #539132) per liter of culture. Tangential flow filtration, followed by centrifugation produced cell pellets for the mass-spectrometry analysis. All samples were kept at −80°C until analysis.

### Messenger RNA Preparation

*Ca.* Pelagibacter ubique cells used in microarray experiments were grown in batch cultures as described above. Cells (40 ml for each biological replicate) were collected via centrifugation, and RNA was extracted using RNeasy Mini kits (Qiagen), followed by amplification with MessageAmp-II Bacteria RNA amplification kit (Ambion). The resulting aRNA was then screened for length and quality using a Bioanalyzer 2100 (Agilent) and quantified utilizing a Nanodrop 1000 spectrophotometer (Thermo Fisher Scientific). 5.5 µg of biotinylated aRNA from each sample was then fractionated and hybridized (45°C) overnight to custom *Ca.* Pelagibacter ubique Affymetrix GeneChip arrays that contained probes for strains HTCC1002, HTCC1062 and HTCC7211 (Pubiquea520471f) using Affymetrix GeneChip Fluidics Station 450, and Affymetrix GeneChip Hybridization Oven 640. Arrays were then washed as per the manufacturer's instructions and the resulting images were analyzed using an Affymetrix GeneChip Scanner 3000. Fluorescence measurements were normalized over all 18 microarray chips.

### Microarray Clustering

A modified radial coordinate visualization plot was used for illustrating mRNA expression in a manner that accentuated condition-specific preferential transcription. In Figure 2, dimensional anchors (DA) representing each of the six microarray samples were positioned manually around the circumference of a circle such that iron-limited samples are on the left, samples with excess iron are on the right, and the vertical placement corresponds to the culture's transition from exponential growth (bottom) to stationary phase (top). Each gene is represented by a single point, positioned according to the relative abundances between every sample pair, and sized according to the largest observed change in expression level. *P* *T* *_g_* is the point for gene *g*, with attributes *x*, *y*, and *s* describing its x-axis position, y-axis position, and size, respectively. *S_i,g_* is the log base-10 average fluorescence for gene *g* in sample *i*. *DA_i_* is sample *i*'s dimensional anchor positioned at (*DA_i,x_*, *DA_i,y_*) on the graph.
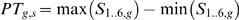


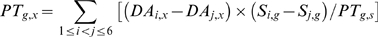


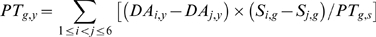
This type of graph is ideal for revealing if a given gene's transcript abundance is changing as a result of iron limitation or as a result of the stationary phase transcriptome remodeling induced by iron limitation.

### Global TFE Protein Preparation

Four samples were prepared using the TFE (2,2,2-Trifluoroethanol) digestion method. The cell pellets were reconstituted in 100 mM NH_4_HCO_3_, pH 8.4 buffer and transferred to a siliconized 0.6 mL microcentrifuge tube. 0.1 mm Zirconia/Silica Beads were added to the top of the tube and bead beat at maximum speed for 3 minutes and immediately placed on ice. A hole was poked in the base of the 0.6 mL siliconized eppendorf tube and placed in a 1.5 mL siliconized eppendorf tube. The sample was then centrifuged for 5 minutes at 14,000 rpm at 4°C. The cell lysis was mixed to a homogenized state and the volume was determined using a pipette. The sample concentration was determined with a Coomassie protein assay and read on a microplate reader. TFE was added to a concentration of 50%. The sample was then homogenized by sonication for one minute in an ice bath followed by incubation at 60°C for two hours with gentle shaking (300 rpm). Proteins were reduced by adding DTT to a final concentration of 2 mM, sonicated for one minute in an ice bath and incubated at 37°C for one hour with gentle shaking. Samples were then diluted 5-fold with 100 mM NH_4_HCO_3_ to reduce the salt concentration, and CaCl_2_ was added to a final concentration of 1 mM. The sample was digested for 3 hours with Trypsin (Promega, Madison WI) at 37°C at a concentration of 1 unit trypsin/50 units protein. After trypsin incubation, a BCA protein assay was performed on the sample to determine the final concentration and vialed for mass spectrometer analysis.

### Capillary LC-MS Analysis

The custom HPLC system was configured using 65-mL Isco Model 65D syringe pumps (Isco, Inc., Lincoln, NE), 2-position Valco valves (Valco Instruments Co., Houston, TX), and a PAL autosampler (Leap Technologies, Carrboro, NC), allowing for fully automated sample analysis across four separate HPLC columns. Reversed-phase capillary HPLC columns were manufactured in-house by slurry packing 3-µm Jupiter C18 stationary phase (Phenomenex, Torrence, CA) into a 70-cm length of 360 µm o.d.×75 µm i.d. fused silica capillary tubing (Polymicro Technologies Inc., Phoenix, AZ) that incorporated a 0.5-µm retaining screen in a 1/16” custom laser-bored 75 µm i.d. union (screen and union – Valco Instruments Co., Houston, TX; laser bore - Lenox Laser, Glen Arm, MD). Mobile phases consisted of 0.2% acetic acid and 0.05% TFA in water (A) and 0.1% TFA in 90% acetonitrile/10% water (B). The mobile phase flowed through an in-line Degassex DG4400 degasser (Phenomenex, Torrance, CA). The HPLC system was equilibrated at 10 k psi with 100% mobile phase A. Fifty minutes after sample injection the mobile phase was switched to 100% B, which created a near-exponential gradient as mobile phase B displaced A in a 2.5 mL active mixer. A 30-cm length of 360 µm o.d.×15 µm i.d. fused silica tubing was used to split ∼20 µL/min of flow before it reached the injection valve (5 µL sample loop). The split flow controlled the gradient speed under conditions of constant pressure operation (10 k psi). Flow through the capillary HPLC column when equilibrated to 100% mobile phase A was ∼400 nL/min.

MS analysis was performed using a ThermoFinnigan LTQ-Orbitrap mass spectrometer (Thermo Scientific, San Jose, CA) with electrospray ionization (ESI). The HPLC columns were coupled to the mass spectrometer by using an in-house manufactured interface. Chemically etched electrospray emitters, 150 um o.d.×20 um i.d, were used [70]. The heated capillary temperature and spray voltage were 200°C and 2.2 kV, respectively. Data was acquired for 100 min, beginning 65 min after sample injection (15 min into gradient). Orbitrap spectra (AGC 1×106) were collected from 400–2000 m/z at a resolution of 100k followed by data dependant ion trap MS/MS spectra (AGC 1×104) of the six most abundant ions using a collision energy of 35%. A dynamic exclusion time of 60 sec was used to discriminate against previously analyzed ions. Three technical replicates were run on the mass spectrometer for each cell pellet.

### Quantitative Proteomics

Quantitative estimates of peptide abundances, calculated from the area under the isotopic profile, were obtained by using a previously developed accurate mass and time (AMT) tag library [34] to search the mass spectra generated by the 12 runs for the four samples. After deisotoping and calculating monoisotopic mass, mass spectrometric features were matched to database peptides with a mass tolerance window of +/−6ppm and an elution time window of +/−0.1% after alignment in both dimensions. Peptide abundances were reported for those which had observations in at least 2 of the 3 technical replicates. Linear regression normalization was used to normalize each set of technical replicates as described elsewhere [71]. Briefly, the abundance of peptide *x* in sample *i* was transformed into minus versus average space using the following formulas:
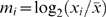


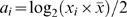
Next, the transformed value was corrected based on a linear regression:
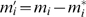
where m_i_* is the value for *m_i_* calculated from the *m* vs *a* regression equation. Lastly, the computed values were deconvoluted to yield the normalized abundances:
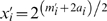
Peptides were excluded from further analysis if the standard deviation exceeded the average measurement value among the three technical replicates for a sample. A final filter was applied to exclude the lowest third of peptides for a given protein, when sorted by the peptides' maximum PeptideProphet F-Score.

Protein abundance was calculated only if a protein had three or more peptides which passed the above filters. Calculating the difference in protein abundance between two samples was a three step process. First, the three replicate peptide abundance measurements were averaged together. Next, the peptide average from sample 1 was divided by the peptide average from sample 2, then log_10_ transformed. Finally, all log_10_ peptide ratios from the same protein were averaged together.

To represent the likelihood that a protein was equally abundant in both samples, the multiple peptide measurements were combined into a single statistic as previously described [72]. Briefly, p-values for individual peptides were calculated using a one-tailed Student's t-test on the technical replicates' *x′* values. A two-tailed Student's t-test was not used because p-values reflecting a large increase would be indistinguishable from p-values reflecting a large decrease. Instead, peptides which changed in the opposite direction from the protein average were assigned a p-value of 1 for their one-tailed Student's t-test. All peptide p-values for a single protein were then combined into a single chi-square statistic using Fisher's method:
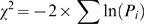



## Supporting Information

Table S1 Coordinates of All Genes Plotted in Figure 2.
(0.25 MB XLS)
